# Better Animal Feeding for Improving the Quality of Ruminant Meat and Dairy

**DOI:** 10.3390/foods10051076

**Published:** 2021-05-13

**Authors:** Manuel Delgado-Pertíñez, Alberto Horcada

**Affiliations:** Departamento de Ciencias Agroforestales, Escuela Técnica Superior de Ingeniería Agronómica, Universidad de Sevilla, Ctra. Utrera km 1, 41013 Sevilla, Spain; albertohi@us.es

The quality of meat and dairy products can be evaluated from the perspective of the farmer seeking high yields and profits or the consumer for whom sensory characteristics are the most important, although health and ethical aspects, such as animal welfare and the environmental impact of the production system, are increasingly becoming concerns worldwide. Animal nutrition is one of the most important environmental factors that significantly influences the quality of meat, milk, and other dairy products; therefore, emphasis is often placed on improving the quality of feed. A main target for improving the nutritional characteristics of meat and dairy is the enhancement of lipid quality, which can be achieved by increasing the content and improving the composition of beneficial fatty acids (FAs)—n-3 polyunsaturated FA (PUFA) and conjugated linoleic acid (CLA)—and decreasing the n-6:n-3 PUFA ratio. Factors such as the forage:concentrate ratio, dietary fat supplements, pasture, etc. have a crucial effect on dairy and meat quality. Some studies have shown that meat and dairy from ruminants grazing on pasture are enriched with bioactive substances of natural origin like phenolic compounds, fat soluble vitamins, terpenes, and lipid components. These animals are also able to consume increasing amounts of by-products or ‘unconventional’ animal feedstuffs, which can improve the health-giving properties of products. In addition, dietary manipulations favouring polyunsaturated FA in dairy and meat lipids increase the risk of lipoperoxidation, which can be efficiently prevented by the use of antioxidants in the diet. Furthermore, the search for biomarkers that link the composition of animal products to livestock feed has become a target of scientific research; these biomarkers allow for traceability from farm to fork, based on the herbivore diet and geographical origin. In this context, the Special Issue ‘Better Animal Feeding for Improving the Quality of Ruminant Meat and Dairy’ aims to provide an integrated analysis of the major aspects of the nature and composition of ruminant meat and dairy (i.e., FA profile, antioxidants, vitamins, muscle:fat ratio, flavor, etc.) and the effect of better animal feeding on the improvement of nutritional and sensory qualities and functional properties beneficial to human health. This Special Issue comprises seven valuable works of original research on product quality; four studies were conducted on pasture-based systems (two on sheep cheese quality, one on cow’s milk quality, and one on beef quality), two on alternative feedstuff-based systems (goat dairy product quality), and one on conventional concentrate-fed animals (beef quality).

Of the articles on pasture-based systems, three compared grass-fed regimes with conventional concentrate-fed regimes. Serrapica et al.’s [1] study on a traditional pecorino cheese associated with management and feeding-system seasonality was conducted in two mountain dairy sheep farms rearing the native Bagnolese breed using an outdoor, pasture-based system from April to October (fed with pasture integrated with hay and concentrate mixture) and an indoor system during the rest of the year (fed with hay and cereal grains). The pasture-based (outdoor management system) cheese had higher percentages of unsaturated FAs (UFA), C18:3, and rumenic (CLA cis-9, trans-11) and vaccenic (C18:1 trans-11) acids, and lower percentages of C14:0 and C16:0, along with a reduction in the atherogenic index. The pasture-based cheeses also displayed higher intensities of almost all sensory attributes, including odour, flavour, taste, and texture descriptors. Optimisation of farm production includes the rational use of forages and respect for the environment, and offers the best quality of products to consumers. Therefore, De la Torre-Santos et al. [2] investigated the effect of the mode of supply of grass to dairy cows (grazing, cut-and-carry, zero-grazing, or grass silage) on milk performance and milk antioxidant and FA profiles to identify biomarkers of the feeding systems. Changes in milk yield and composition were associated with the feeding system. The grazing system promoted a higher dry matter intake and greater milk yield as well as a higher proportion of UFA (with significant differences in the proportion of vaccenic and rumenic acids) and lutein than the grass silage and zero-grazing systems. The authors propose the 18:1 trans-11 to 18:1 trans-10 ratio as a biomarker to identify the milk produced by cattle in the grazing system. The third study [3] comparing feeding regimes investigated beef quality. The primary aim of this study was to determine the influence of two organic production systems—organic grass-fed and organic concentrate-fed—compared to a conventional concentrate-fed system, on the evolution of texture properties and histological attributes of muscle fibres during the ageing process of the meat of the Retinta breed. Although the meat of organic grass-fed animals was initially tougher, its speed of tenderisation was higher in the first ageing days than that of meat from concentrate-fed systems. In all systems of feeding, sarcomere length increased during the ageing period, and showed a negative correlation with shear force, which is related to the tenderising of the meat. Finally, the fourth study on pasture-based systems [4] is the first nutritional analysis of dairy products from the traditional Roja Mallorquina sheep breed raised on a part-time grazing system, which could support the implementation of strategies to promote their commercialisation and obtain product labelling as ‘pasture-fed’ or other specific marks. Results showed that fresh soft cheese, compared to the original milk and ripened cheese, was generally characterised by better nutritional value for human health according to the fat-soluble components—a favourable level of retention of retinol and α-tocopherol and a lower percentage of saturated FAs (SFA) and lower atherogenic (AI) and thrombogenic (TI) indices. Furthermore, acetoin and products of lactose and citrate metabolism played an important role in the development of the aromatic attributes of both kinds of cheese.

In the Mediterranean region, high amounts of by-products are available that can be used as alternative feedstuffs for ruminants, lowering feed costs, and enhancing farm sustainability, while reducing the environmental impact of livestock production. Monllor et al. [5] determined the effect of three levels of inclusion of by-product silages (artichoke plants and broccoli by-product) in goat diets on milk yield and quality. They proposed that the threshold level of inclusion of by-products in diets, without negative effects on milk yield and composition and the metabolic status of the animals, would be 40% of the dietary dry matter. From the point of view of human health (AI and TI), the study demonstrated that the inclusion of artichoke plant silage in the animals’ diet improved the milk lipid profile compared to broccoli silage, due to a lower SFA content (C12:0, C14:0, and C16:0) and a higher PUFA concentration, especially of vaccenic and rumenic acids, without any differences from the control treatment. Spain is the primary producer of oranges in the European Union and the sixth largest global producer; orange pulp, the principal by-product, can partially replace cereal grains in ruminant feedstuffs. Therefore, Guzmán et al. [6] evaluated the influence of diets with dry orange pulp pellets on the physicochemical characteristics, sensory properties, and volatile compound profiles of cheeses traditionally made from the milk of the Payoya breed of goat. Results showed that dried citrus pulp can be used in the goat diet as a substitute for cereal in concentrates because it did not substantially affect the distinctive final characteristics of the ripened raw milk cheeses.

In the last paper of this Special Issue, Barahona et al. [7] studied the effect of two feeding systems based on concentrate and maize silage and two packaging systems, vacuum and modified atmosphere, on the quality of meat from the Avileña breed of cattle. Their results showed that animals fed with concentrate had higher carcass weight; however, the use of maize silage improved the tenderness of meat and the FA content. In general, vacuum-packed meat from maize silage-fed animals was the most preferred by consumers. An alternative feeding system based on the use of maize silage may improve some aspects of meat quality.

In summary, the Special Issue provides evidence that production systems based on grass and forages produce healthier products with better sensory attributes, thereby promoting the consumption of healthier foods. At the same time, as suggested by several authors, high amounts of by-products can be used as alternative feedstuffs for ruminants, enhancing farm sustainability while improving the health-giving properties of products.

## References

[1] Serrapica F., Masucci F., Di Francia A., Napolitano F., Braghieri A., Esposito G., Romano R. (2020). Seasonal Variation of Chemical Composition, Fatty Acid Profile, and Sensory Properties of a Mountain Pecorino Cheese. Foods.

[2] De La Torre-Santos S., Royo L.J., Martínez-Fernández A., Chocarro C., Vicente F. (2020). The Mode of Grass Supply to Dairy Cows Impacts on Fatty Acid and Antioxidant Profile of Milk. Foods.

[3] García-Torres S., López-Gajardo A., Tejerina D., Prior E., de Vaca M.C., Horcada A. (2020). Effect of Two Organic Production Strategies and Ageing Time on Textural Characteristics of Beef from the Retinta Breed. Foods.

[4] Gutiérrez-Peña R., Avilés C., Galán-Soldevilla H., Polvillo O., Ruiz Pérez-Cacho P., Guzmán J.L., Horcada A., Delgado-Pertíñez M. (2021). Physicochemical Composition, Antioxidant Status, Fatty Acid Profile, and Volatile Compounds of Milk and Fresh and Ripened Ewes’ Cheese from a Sustainable Part-Time Grazing System. Foods.

[5] Monllor P., Romero G., Atzori A.S., Sandoval-Castro C.A., Ayala-Burgos A.J., Roca A., Sendra E., Díaz J.R. (2020). Composition, Mineral and Fatty Acid Profiles of Milk from Goats Fed with Different Proportions of Broccoli and Artichoke Plant By-Products. Foods.

[6] Guzmán J.L., Delgado Pertíñez M., Galán Soldevilla H., Ruiz Pérez-Cacho P., Polvillo Polo O., Zarazaga L.Á., Avilés Ramírez C. (2020). Effect of Citrus By-product on Physicochemical Parameters, Sensory Analysis and Volatile Composition of Different Kinds of Cheese from Raw Goat Milk. Foods.

[7] Barahona M., Hachemi M.A., Olleta J.L., González M.d.M., Campo M.d.M. (2020). Feeding, Muscle and Packaging Effects on Meat Quality and Consumer Acceptability of Avileña-Negra Ibérica Beef. Foods.

